# The effects of ageing and adrenergic challenge on electrocardiographic phenotypes in a murine model of long QT syndrome type 3

**DOI:** 10.1038/s41598-017-11210-3

**Published:** 2017-09-11

**Authors:** Karan R. Chadda, Shiraz Ahmad, Haseeb Valli, Ingrid den Uijl, Ali BAK Al-Hadithi, Samantha C. Salvage, Andrew A. Grace, Christopher L.-H. Huang, Kamalan Jeevaratnam

**Affiliations:** 1 Physiological Laboratory, University of Cambridge, Downing Street, Cambridge, CB2 3EG United Kingdom; 2 Faculty of Health and Medical Sciences, University of Surrey, Guildford, GU2 7AL United Kingdom; 3 School of Medicine, Perdana University - Royal College of Surgeons Ireland, 43400 Serdang, Selangor, Darul Ehsan Malaysia; 4 Division of Cardiovascular Biology, Department of Biochemistry, University of Cambridge, Hopkins Building, Cambridge, CB2 1QW United Kingdom; 5 Department of Biochemistry, University of Cambridge, Hopkins Building, Cambridge, CB2 1QW United Kingdom

## Abstract

Long QT Syndrome 3 (LQTS3) arises from gain-of-function Na_v_1.5 mutations, prolonging action potential repolarisation and electrocardiographic (ECG) QT interval, associated with increased age-dependent risk for major arrhythmic events, and paradoxical responses to β-adrenergic agents. We investigated for independent and interacting effects of age and S*cn5a*+*/ΔKPQ* genotype in anaesthetised mice modelling LQTS3 on ECG phenotypes before and following β-agonist challenge, and upon fibrotic change. Prolonged ventricular *recovery* was independently associated with S*cn5a*+*/ΔKPQ* and age. Ventricular *activation* was prolonged in old S*cn5a*+*/ΔKPQ* mice (p = 0.03). We associated S*cn5a*+*/ΔKPQ* with increased atrial and ventricular fibrosis (both: p < 0.001). Ventricles also showed increased fibrosis with age (p < 0.001). Age and S*cn5a*+*/ΔKPQ* interacted in increasing incidences of repolarisation alternans (p = 0.02). Dobutamine increased ventricular rate (p < 0.001) and reduced both atrioventricular conduction (PR segment-p = 0.02; PR interval-p = 0.02) and incidences of repolarisation alternans (p < 0.001) in all mice. However, in S*cn5a*+*/ΔKPQ* mice, dobutamine delayed the changes in ventricular repolarisation following corresponding increases in ventricular rate. The present findings implicate interactions between age and S*cn5a*+*/ΔKPQ* in prolonging ventricular activation, correlating them with fibrotic change for the first time, adding activation abnormalities to established recovery abnormalities in LQTS3. These findings, together with dynamic electrophysiological responses to β-adrenergic challenge, have therapeutic implications for ageing LQTS patients.

## Introduction

Long QT Syndrome (LQTS) is characterised by prolonged electrocardiographic (ECG) QT intervals reflecting increased ventricular action potential durations (APD) and is associated with increased incidences of arrhythmogenesis. The long QT syndrome 3 (LQTS3) variant specifically arises from gain-of-function mutations in the inactivation domain of the *SCN5A* gene that encodes the cardiac Na^+^ channel, Na_v_1.5^1–3^. These gain-of-function mutations result in recovery abnormalities attributable to increased late Na^+^ current (*I*_Na-L_) known to enhance both triggers and substrates for arrhythmogenesis. *Arrhythmic triggering* arises from an occurrence of afterdepolarisation events during or immediately following an AP. *Arrhythmic substrate* arises from re-entrant processes re-exciting recovered regions resulting from the increased *I*_Na-L_^4^.

The aim of the present study to investigate the effects of age and adrenergic challenge on the LQTS3 phenotype was prompted by previous clinical and experimental work. Firstly, there is continued discussion concerning effects of effects of ageing in LQTS^5^ wherein human studies report reduced ages of onset (~50 ± 14 years) for atrial fibrillation (AF)^6^. More specifically in LQTS3, (a) LQTS3 genotypes were powerful predictors of fatal or near-fatal events after age 40, even compared to LQTS2 and LQTS1 genotypes, with similar risks associated with each LQTS genotype when excluding patients treated with β-blockers from the analysis^7^. (b) The specific association of LQTS arrhythmias occurring after 40 years of age with LQTS3 phenotypes^8^. (c) Complementary experimental studies showing increased arrhymogenicity with age in murine models of LQTS3^9^.

Secondly, there are particular clinical paradoxical adrenergic effects in LQTS3. On the one hand, exercise and fright precipitate acute accentuations of QT interval prolongation, T-wave alternans and torsade de pointes accounting for 32% of fatal cardiac events in LQTS3^10, 11^. Adrenergic provocation similarly provokes ECG T wave lability associated with cardiac arrest or syncope in some LQTS3 patients^12^. β blockade reduces risks of cardiac events specifically in female LQTS3 patients, although the effect in males is yet to be determined due to insufficient data^13^. On the other hand, in contrast to LQTS1 or LQTS2 patients, 39% of LQTS3 fatalities occur at rest or during sleep, when adrenergic tone is expected to be low and cholinergic activity is expected to be high^11^,^14^. A further clinical study associates higher incidences of cardiac events with both the LQTS3 and LQTS2 genotype amongst a cohort of LQTS patients treated with β blockers^15^. Similarly, contrasts between adrenergic effects have also been shown in experimental studies. Thus, β-adrenergic stimulation was variously pro-arrhythmic^16^, anti-arrhythmic^17, 18^ or without effect in LQTS3 models^19^. Furthermore, β -blockade was also shown to reduce arrhythmogenicity^16, 20^ and triggering events^20^. Yet other studies showed β -blockade accentuated reentrant substrate^19^ and exacerbated transmural gradients of repolarisation with potential pro-arrhythmic effects^20^.

The present ECG and histological experiments explored the physiological basis for the above effects for the first time, using the *Scn5a*+*/ΔKPQ* murine model for LQTS3. This model carries a deletion of three conserved amino acids, Lys 1505, Pro 1506 and Gln 1507, in the Na_v_1.5 III–IV linker responsible for fast inactivation, known to correspond to a particularly severe clinical LQTS3 phenotype^21, 22^. Previous studies reported that the *Scn5a*+*/ΔKPQ* murine model recapitulated many clinical features of LQTS3, including its ECG abnormalities and arrhythmic tendency^18, 19, 23^. Previous work had demonstrated the mechanistic basis of possible arrhythmic phenotypes in studies demonstrating Na^+^ current abnormalities^19^, triggering events^20, 24^ and arrhythmic substrate^20, 25^. The present work uses electrocardiographic and histological studies to examine for independent and interacting effects of ageing and *Scn5a*+*/ΔKPQ* genotype on arrhythmic phenotype and electrophysiological properties, before and following β1-adrenergic challenge, and the contribution of fibrotic change to these effects, in intact animals for first time.

## Materials and Methods

### Animals

A total of 12 wild-type (WT) and 13 *Scn5a*+*/ΔKPQ* S129sv mice were studied. They were housed in an animal facility maintained at 21 °C with 12 h light/dark cycles, fed sterile chow (RM3 Maintenance Diet; SDS, Witham, Essex, UK) and had free access to water. All experimental protocols were approved under the UK Home Office regulations (Animals (Scientific Procedures) Act 1986 Amendment Regulations 2012) following ethical review by the University of Cambridge Animal Welfare and Ethical Review Body (AWERB) and conducted under a designated project license. The experiments also conformed to the Guide for the Care and Use of Laboratory Animals, U.S. National Institutes of Health (NIH Publication No. 85-23, revised 1996). The WT and *Scn5a*+*/ΔKPQ* mice were each further divided into young (3 ± 1 month) and old (12 ± 1 month) groups.

### Electrocardiographic analysis

For the electrocardiographic (ECG) study, the mouse groups consisted of young WT (n = 7), old WT (n = 5), young *Scn5a*+*/ΔKPQ* (n = 7) and old *Scn5a*+*/ΔKPQ* mice (n = 6). The ECG recordings followed a previously published approach^26^. To anaesthetise each mouse, avertin (2,2,2 trimethylethanol, Sigma-Aldrich, Poole UK) was administered into the intra-peritoneal space before the ECG recordings were made. Avertin is known to have fewer cardiovascular and electrophysiological conduction effects than other anaesthetic agents, such as isoflurane and xylazine-ketamine^27^. For example, previous work indicated that ketamine provides reliable relaxation, sedation, and analgesia in mice but exerts bradycardic effects^28^. All measurements were consistently performed after a 5-min period following establishment of anaesthesia and the recording setup in an attempt to allow autonomic levels to stabilise. The mice were left undisturbed in a dark box until the sedation took effect, weighed, and then placed in a supine position on a heated platform to maintain a 37 °C body temperature. Four 2-mm diameter electrodes (MLA1204; AD Instruments, Colorado Springs, CO, USA) were placed in the limbs and connected to a 4-channel NL844 pre-amplifier. The outputs were then led through 4-channel NL820 isolator and NL135 low-pass filter units (set at a 1.0-kHz cut-off and with a 50-Hz notch) within a NL900D chassis and power supply (Neurolog-Digitimer, Hertfordshire, UK). The signal was sampled at 5 kHz and analogue-to-digital conversion employed a CED 1401c interface (Cambridge Electronic Design, Cambridge, UK). This then conveyed Lead II ECG traces to a computer for display and recording using Spike II software (Cambridge Electronic Design).

**Figure 1:**
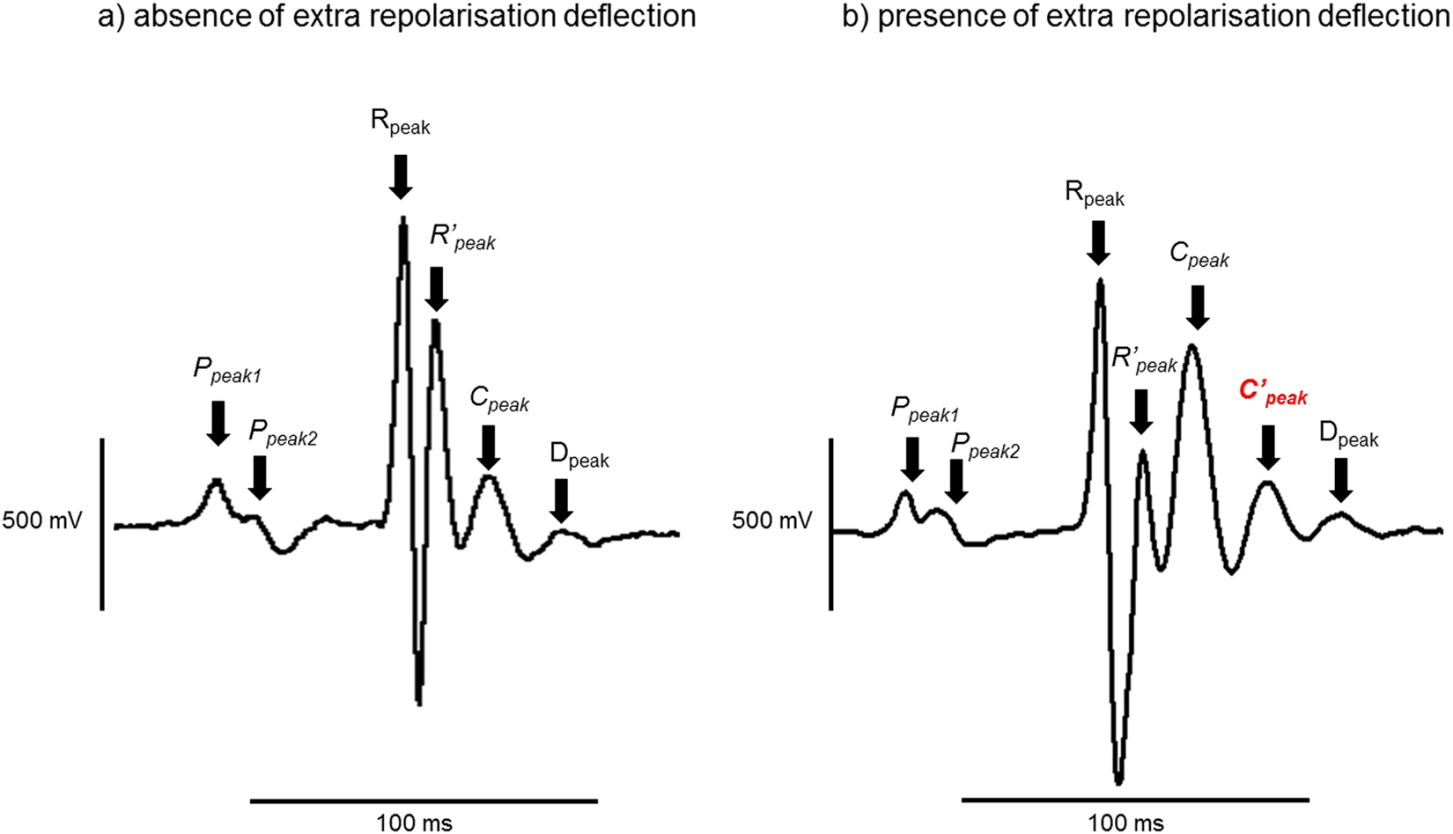
Representative peaks in the ECG records All peaks shown here, apart from C’_peak_, marked in red, were consistent across all the ECG records. C’_peak_ was absent in some ECG records (**a**) but present in others (**b**). The cursors were then applied accordingly to encompass this difference, by including a QD duration, while allowing the same intervals to be analysed across all records.

**Figure 2:**
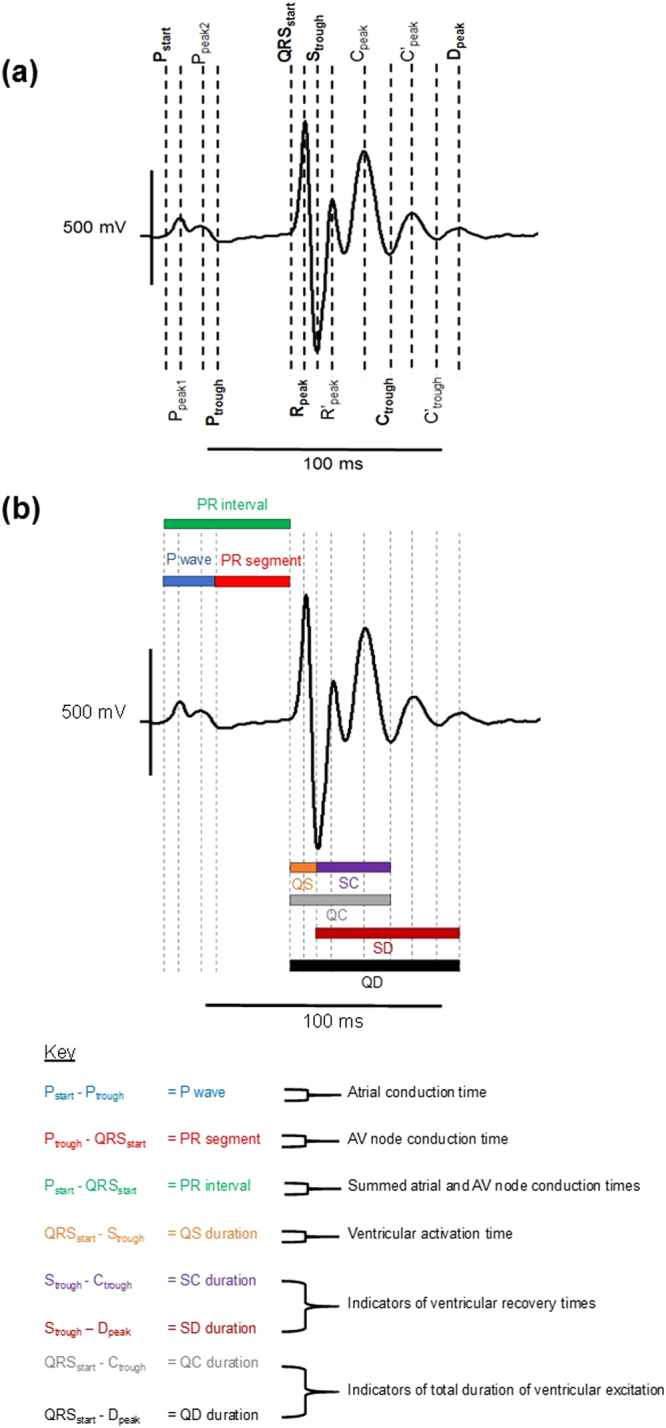
Schematic representation of the ECG analysis method (**a**) This representative lead II ECG trace obtained from an avertin anaesthetized mouse illustrates the cursors (dashed vertical lines) that were applied using Spike II software (Cambridge Electronic Design). Bipolar recordings were obtained with the negative electrode placed on the right forelimb and the positive electrode placed on the left hindlimb. The cursors indicate key reference features in the observed ECGs. Those marked in bold type were the key parameters sought in the automated analysis. Those marked in standard type indicate consistent features in the ECG traces that were used in the signal analysis programme to determine those key parameters. (**b**) This same example lead II ECG trace now has the intervals sought in the automated analysis programme superimposed. The key below the trace shows the physiological relevance of each of those ECG intervals. This analysis yielded the following ECG intervals which provide indications of different aspects of electrophysiological function: RR interval (the ventricular rate reflecting sino-atrial node pacemaker function); P wave duration (atrial conduction time); PR segment duration (atrioventricular (AV) node conduction time); PR interval (combined atrial and AV node conduction times); QS interval (ventricular activation time); SC and SD intervals (indicators of ventricular recovery time); QC and QD intervals (indicators of the total duration of ventricular excitation).

Baseline ECGs were recorded for 10- to 15-min, including a 5-min stabilisation period. Dobutamine hydrochloride (0.3 mg/kg: Sigma Aldrich, Poole UK) was then injected intraperitoneally. The ECG recordings continued for at least another 10-min after an observable effect on the ECG trace. Baseline stability in the ECG recordings was optimised by securing the limbs of the mice with adhesive tape to minimize movement artefacts and by performing experiments within a grounded Faraday cage to reduce electrical noise. The ECG record analysis also employed Spike II software and no digital filters were applied during the analysis. The ECG deflections ended with a “D peak” (Fig. 1a), which in some records could be preceded by an additional “*C’ peak*” (Fig. 1b) before final return of the trace to the isoelectric baseline. All the remaining peaks and troughs were consistent in their occurrence through all the experimental groups that were studied. Thus, there was an internally consistent general ECG waveform, meaning that no traces were excluded. This allowed the application of cursors at the peaks and troughs of the trace as shown in the representative example in Fig. 2a. Only Lead II ECG traces were analysed. Lead I traces were used as references to guide application of cursors. The cursors indicate key reference features in the observed ECGs and were used to subsequently measure interval durations (Fig. 2b).

This analysis yielded the following ECG intervals which provide indications of different aspects of electrophysiological function: RR interval (the ventricular rate reflecting sino-atrial node pacemaker function); P wave duration (atrial conduction time); PR segment duration (atrioventricular (AV) node conduction time); PR interval (summed atrial and AV node conduction times); QS interval (ventricular activation time); SC and SD intervals (indicators of ventricular recovery time); QC and QD intervals (indicators of the total duration of ventricular excitation). Earlier studies comparing recovery intervals at different heart rates used Bazett’s formula^29^ with an adjustment for mice: (QT/√(RR/100)^30^. However, the intervals analysed in the present study used uncorrected values. Thus recent reports pointed out that the modified Bazett’s formula can underestimate the QT interval and introduce systematic measurement errors in murine hearts, particularly for recovery diseases such as LQTS^31–33^

The data from the cursor time points was then imported into and analysed with custom-written software in the open-source R programming language (R Core Team, 2015). This was used to calculate the mean and standard errors of the desired ECG interval durations and to quantify the presence of C_peak_ alternans. Analysis was performed on 300-sec of ECG data immediately prior to administration of dobutamine and 300-sec post-dobutamine period after having an observable effect. This quantitative analysis was only applied to parts of the trace showing a steady-state ventricular rate.

### Histological analysis

The quantification of cardiac fibrosis followed methods used in previous studies in the *Scn5a*+/− mouse model and therefore permitted the present results to be compared with the previous observations^34^. The mice were killed by cervical dislocation and the hearts dissected out. Custom made Krebs buffer (containing, in mM, NaCl 119, NaHCO_3_ 25, KCl 4, KH_2_PO_4_ 1.2, MgCl_2_ 1, CaCl_2_ 1.8, glucose 10 and Na-pyruvate 2, pH 7.4, 95% O_2_/5% CO_2_; British Oxygen Company, Manchester, UK) was used to flush the isolated hearts, which were then perfused with 4% buffered formalin for 5-min before being kept in formalin overnight. Following this fixation process, gross transverse sections were taken. This was followed by routine tissue processing and paraffin embedding. The 7 μm thick sections were then stained using a Sirius red protocol, involving a series of immersions: xylene for 2-min; new batch of xylene for 2-min; 95%, 70% and 50% ethanol for 2-min each; Weigerts Haemotoxylin for 8-min; running water for 10-min; picro-sirius red solution for 1 hour; acidified water for 16 dips; 3 changes of 100% ethanol for 1-min each and xylene for 3 dips. The slides were then mounted and subsequently viewed, magnified, and digitally acquired using the Nano Zoomer 2.0 Digital Pathology system (Hamamatsu, Hertfordshire, UK).

Following magnification, a custom-made 17 cm × 30 cm morphometric grid, consisting of square boxes of dimension 1 cm × 1 cm, corresponding to an approximate 0.2 mm × 0.2 mm area of tissue, was then superimposed on each photomicrograph. If a square occupied either completely or partially by cardiac tissue showed the presence of fibrosis, it was counted and then the number of these squares was then expressed as a percentage of total cardiac tissue area for each heart. This analysis was carried out blindly by two investigators independently and then their results were compared for consistency by applying an inter-class correlation coefficient analysis (ICC) to identify if both assessors were in agreement with what counted as fibrotic change. ICC can be interpreted as follows: 0–0.2 indicates *poor* agreement: 0.3–0.4 indicates *fair* agreement; 0.5–0.6 indicates moderate agreement; 0.7–0.8 indicates *strong* agreement; and >0.8 indicates *almost perfect* agreement.

### Statistical analysis

Statistical analyses used Stata SE14 (StataCorp, TX, USA). Each ECG feature or histological parameter was analysed as a separate outcome using regression analysis. We adopted numbers of replicates, n, for each experimental group, that were sufficient for this multivariable regression analysis. Although there are 3 variables (age, genotype and dobutamine challenge), our study involved paired comparisons and was confined to exploring two (and not three) way interactions between variables. We then emphasised only clear and significant associations, reporting conservatively on associations that were narrowly significant, and indicated instances where there was insufficient evidence to support an interaction, rather than inferring its absence. The choice of regression model was determined by the distribution of the parameter under analysis. Thus, the ECG intervals were analysed with multilevel linear regression. Histology results were analysed with a linear regression. The C_peak_ alternans parameters included the number of incidences of an alternans episode and the total number of beats involving alternans. Both were analysed with a multilevel negative binomial regression. QC lag and QD lag were dichotomised into an absence or presence of such lag and analysed with a logistic regression.

To analyse the association between ECG features with dobutamine treatment, age and genotype, regression analyses were conducted in two parts. First a univariable regression analysis was performed to select possible risk factors and confounders. Variables with p < 0.20 were selected for the multivariable analyses. The multivariable regression analysis was performed using backwards elimination. All variables with univariable p < 0.20 were included in the first model and variables with p-value > 0.05 were eliminated from the model. To control for confounding, variables were eliminated one by one from the model. If the coefficients from the remaining variables were changed by >25%, the eliminated variable was then retained in the model as a confounder. Interactions were tested which were thought to be of biological interest. The following interactions were tested: age × genotype, age × treatment and genotype × treatment. Where appropriate, specifically when mice were treated with dobutamine, multilevel analyses were performed to adjust for multiple measurements per mouse.

Positive regression coefficients reflected an increase of the outcome with the named category in comparison with the reference category. Negative coefficients indicated a decrease in the outcome for the named category in comparison to the reference category. The reference categories were *pre-dobutamine challenge* for treatment, *young* for age and *WT* for genotype. A significant interaction means that the risk factor effects are not independent, but are different for various categories. Interpretation of this statistical analysis considered the following limitations. First, a three-way interaction between age, genotype and treatment was not possible due to the available sample size. Secondly, the available sample size and the multiple parameters tested within these needed to consider effects of multiple testing: the larger the numbers of parameters tested, the more likely any given parameter would appear to have an association with at least one category.

## Results

### Qualitative assessment of ECG records

**Figure 3:**
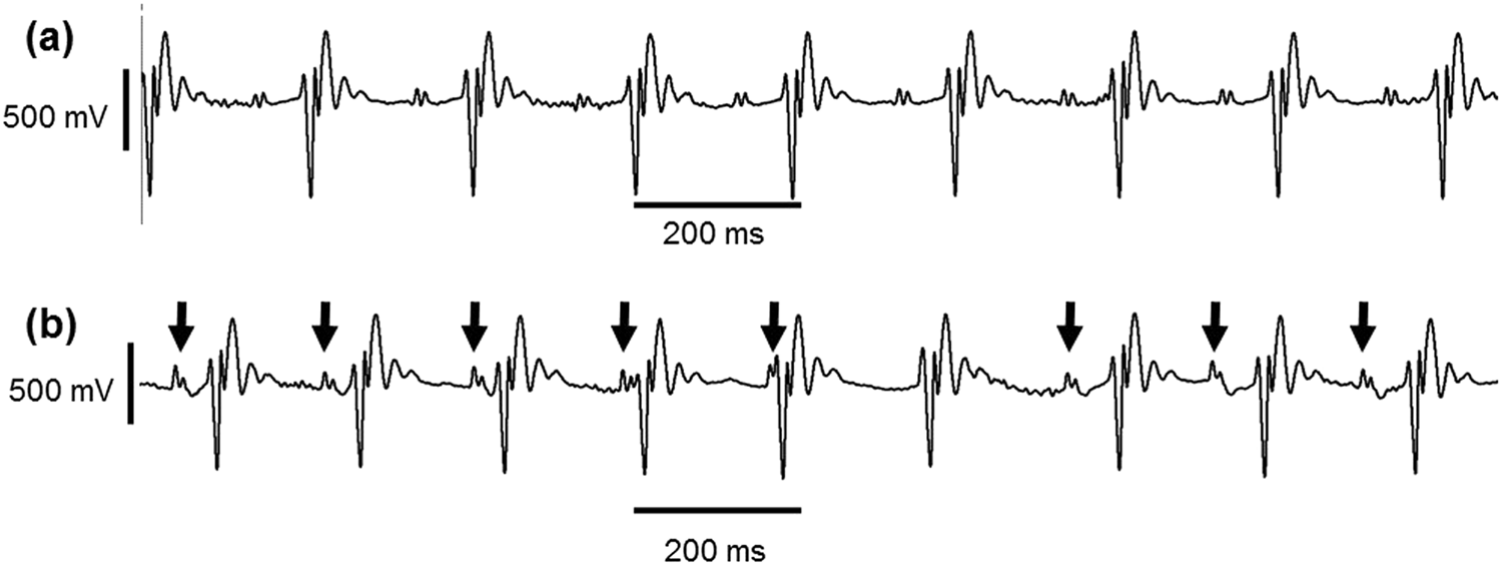
Representative lead II ECG traces following qualitative analysis (**a**) This trace shows a normal sinus rhythm that was seen throughout the whole recordings, pre- and post-dobutamine challenge, in all young WT (n = 7), all old WT (n = 5) and all young *Scn5a*+/ΔKPQ (n = 7) but in 4 out of 6 of the old *Scn5a*+/ΔKPQ. (**b**) This trace shows isorhythmic AV dissociation that was observed in 2 out of 6 old Scn5a+/ΔKPQ mice post-dobutamine. The arrows are pointing to the P waves, of which there is a noticeable absence in the middle part of the trace.

Figure 3 shows examples of different types of ECG lead II waveforms observed in the avertin-anaesthetised mice. The predominant ECG waveform pattern across all groups, whether pre-or post-dobutamine challenge, was one of normal sinus rhythm (Fig. 3a). However, two old *Scn5a*+*/ΔKPQ* mice showed arrhythmic episodes *post-dobutamine challenge*. These patterns were not seen in young *Scn5a*+*/ΔKPQ*, consistent with possible influences of age on arrhythmic tendency. Thus, both of these old *Scn5a+/ΔKPQ* mice showed episodes of isorhythmic AV dissociation (Fig. 3b). During such episodes, the P wave morphology appeared identical to that seen during the previous periods of sinus rhythm and the corresponding mean P–P interval was 178 ms. QRS morphology also appeared unchanged from the earlier periods of the recording and the R–R interval measured 172 ms. However, the PR interval progressively shortened with the P wave eventually merging with the QRS complex, culminating in its disappearance. The arrows in Fig. 3b indicate successive P waves immediately preceding the first five QRS complexes illustrated in the trace, and their disappearance in the next electrocardiographic complex, followed by their return. This return was associated with a prolongation of the R–R interval, with matched P–P and R–R intervals (184 ms) and a fixed PR interval once again (54 ms).

These observations suggested that in response to dobutamine the P wave rate and QRS rate both increased to similar degrees but were intermittently not identical, giving rise to the time shift of the P wave relative to the QRS complex. During such episodes, the QRS morphology continued to appear narrow and unchanged and identical to that earlier in the recording throughout the episode. This was consistent with a high escape focus, most likely junctional. However, the QRS complexes wherein the P wave is merged or absent could not be driven by sinus node impulse. Furthermore, where the QRS complex occurred in the absence of a sinus node input, there was no R–R delay; this makes it unlikely that this was a sinus pause with an accompanying isolated ventricular escape beat. In contrast, the consistent R - R interval during this period suggested that the ventricles were being driven by an ectopic focus.

The features above are thus consistent with intermittent isorhythmic AV dissociation with an accelerated junctional pacemaker rhythm, such as that reported previously. This could reflect some degree of age-related sinus node dysfunction that may have contributed with a delayed tachycardic response thus being usurped by the junctional focus^35, 36^.

### Analysis of ECG features related to sino-atrial, atrio-ventricular and atrial conduction

Table 1 summarises ECG features representing both activation and recovery events in each cardiac cycle in the atria and ventricles. This mean data was obtained from 5-min intervals both pre- and post-dobutamine challenge. Table 2 shows the results of a multilevel linear regression analysis for independent and interactive associations between these ECG features with dobutamine challenge, increased age, and *Scn5a*+*/ΔKPQ* genotype.

**Table 1:** Electrocardiographic parameters.

	Pre-dobutamine challenge				Post-dobutamine challenge			
	WT		*Scn5a*+*/ΔKPQ*		WT		*Scn5a*+*/Δ;KPQ*	
	Young	Old	Young	Old	Young	Old	Young	Old
	(7)	(5)	(7)	(6)	(7)	(5)	(7)	(6)
Sino-atrial node function								
Ventricular rate (Hz)	6.7 ± 0.27	6.59 ± 0.14	6.53 ± 0.13	5.74 ± 0.28	10.17 ± 0.21	8.74 ± 0.19	10.25 ± 0.36	8.71 ± 0.36
Atrial conduction								
P wave duration (ms)	25.59 ± 0.49	25.64 ± 0.92	26.32 ± 0.42	27.26 ± 0.49	25.86 ± 0.40	25.79 ± 0.95	25.55 ± 0.19	28 ± 1.16
Atrio-ventricular node conduction								
PR segment (ms)	17.08 ± 2.50	23.95 ± 2.62	21.51 ± 2.19	23.23 ± 1.19	12.55 ± 1.60	22.61 ± 3.15	13.88 ± 3.76	20.74 ± 2.77
PR interval (ms)	42.72 ± 2.36	49.61 ± 1.77	47.83 ± 1.95	50.59 ± 0.88	38.41 ± 1.87	48.39 ± 2.34	39.43 ± 3.89	48.74 ± 2.55
Ventricular activation								
QS duration (ms)	10.81 ± 0.47	10.51 ± 0.69	11.05 ± 0.39	13.44 ± 0.65	11 ± 0.48	10.33 ± 0.86	11.8 ± 0.58	13.01 ± 0.73
Ventricular recovery								
SC duration(ms)	30.64 ± 0.27	32.8 ± 1.10	32.39 ± 0.81	31.19 ± 0.55	29.65 ± 0.57	31.28 ± 0.70	31.64 ± 0.64	32.73 ± 1.16
SD duration(ms)	41.37 ± 0.37	46.19 ± 3.48	55.41 ± 3.23	58.04 ± 2.82	41.32 ± 0.59	44.91 ± 3.42	49.74 ± 1.73	58.84 ± 3.55
QC duration(ms)	41.45 ± 0.57	43.27 ± 1.41	43.44 ± 0.53	44.63 ± 0.84	40.65 ± 0.83	41.61 ± 1.35	43.45 ± 0.62	45.74 ± 0.89
QD duration(ms)	52.18 ± 0.39	56.67 ± 3.46	66.46 ± 3.24	71.48 ± 3.03	52.32 ± 0.58	55.24 ± 3.10	61.55 ± 1.64	71.86 ± 3.54

**Table 2:** Results of multi-level linear regression of the association between various electrocardiographic features with dobutamine treat.

		Ventricular rate	P wave duration	PR segment	PR interval	QS duration	SC duration	SD duration	QC duration	QD duration
Treatment	Pre-dobutamine	—	—	—	—	—	—	—	—	—
	Post-dobutamine	3.6 (<0.001)	x	−4.3 (0.02)	−4.2 (0.02)	x	x	−1.7 (0.04)	x	x
Age	Young	—	—	—	—	—	—	—	—	—
	Old	−0.48 (0.08)	x	6.3 (0.001)	7.3 (<0.001)	x	x	x	1.6 (0.03)	5.8 (0.002)
Genotype	WT	—	—	—	—	—	—	—	—	—
	*Scn5a*+*/∆KPQ*	x	x	x	x	x	x	12 (<0.001)	2.5 (0.001)	13 (<0.001)
Old × *Scn5a*+*/∆KPQ*		x	x	x	x	2.3 (0.03)	x	x	x	x
Old × post-dobutamine		−1.0 (0.01)	x	x	x	x	x	x	x	x
*Scn5a*+*/∆KPQ* × post-dobutamine		x	x	x	x	x	x	x	x	x

Numbers are regression coefficients with the p-values in brackets, “x” indicates non-significance, “—” indicates a reference category.

Sino-atrial node (SAN) pacemaking increased in frequency following dobutamine challenge in all experimental groups (Table 1). Thus, β-adrenergic stimulation had indistinguishable effects on ventricular rate in *Scn5a*+*/ΔKPQ* compared to WT. In both cases, this effect was diminished in old mice, consistent with a reduced chronotropic competence with age. Thus, ventricular rate, assessed from RR intervals and used as a measure for SAN function, increased following dobutamine challenge (p < 0.001). However, there were no independent effects of either age or genotype on ventricular rates whether before or following dobutamine challenge (Table 2). Nevertheless, age acted as a confounding factor (p = 0.08). Furthermore, age and dobutamine challenge together exerted interacting effects, resulting in old mice showing lower ventricular rates post-dobutamine challenge than young mice (p = 0.01). Finally, genotype exerted no interactive effects with either age or dobutamine challenge on ventricular rate

Dobutamine challenge, age and genotype exerted no independent or interactive effects upon atrial activation as reflected in recorded P wave durations. AV node conduction could be assessed from ECG PR segments intervening between the end of the P wave to the start of the QRS complex. AV node conduction was enhanced by dobutamine challenge, but compromised by age to extents indistinguishable between genotypes. Thus, dobutamine challenge independently decreased (p = 0.02), age independently increased (p = 0.001), but genotype exerted no independent effects on PR segments. There were no interactive associations observed for PR segments. Similar inferences emerged from the analysis of PR intervals, which reflect combined times required for atrial activation and AV conduction. Thus, dobutamine challenge independently decreased (p = 0.02), age independently increased (p < 0.001), but genotype exerted no independent effects on PR intervals. There were no interacting effects involving any of the tested variables on such PR intervals.

### Analysis of ECG features related to ventricular activation and recovery properties

ECG deflections related to the completion of activation and onset of recovery have previously been correlated with the S trough and the R’ peak. We adopted the QS duration to reflect overall ventricular activation in turn resulting from Na^+^ channel activation. Previous reports had suggested that including either the entire, or the late component of the R’ wave, partially captures ventricular repolarisation^31, 37^ overestimating the ventricular activation phase. This finding complements previous reports that had primarily associated LQTS3 only with prolonged repolarisation. Thus, dobutamine challenge, age and genotype exerted no independent effects on QS duration (Table 2). However, age and genotype interacted in increasing QS duration (p = 0.03) with no further interacting effects. Dobutamine challenge did not affect ventricular conduction in contrast to its effects of decreasing PR segment and PR interval, as well decreasing RR interval, in both *Scn5a*+*/ΔKPQ* and WT.

We studied the final electrocardiographic deflections ending each ECG complex as representing the overall duration of electrophysiological activity^38^. Two intervals following the QS deflection, SC and SD durations, were associated with ventricular recovery and were both analysed. The present findings suggest that SC duration may be an indication of the greater part of ventricular recovery towards the resting potential. However, the absence of changes in SC duration, even when comparing the LQTS3 model with WT, suggests that SC duration may not capture the prolonged QT interval attributable to the increased *I*_Na-L_, normally associated with either murine *Scn5a*+*/ΔKPQ* hearts or LQTS3 patients. Thus, dobutamine challenge, age and genotype exerted no independent or interactive effects on SC duration. In contrast, *Scn5a*+*/ΔKPQ* mice showed longer SD durations than WT mice, under all circumstances. This identifies the SD duration with the LQTS3 phenotype and its associated recovery abnormality. This would in turn suggest that the SD duration is inclusive of *I*_Na-L_ contributions to the overall AP duration, typical of LQTS3 phenotypes. Thus, the predominant effect was that genotype independently increased SD duration (p < 0.001) but age had no independent effect. The effects of dobutamine challenge were suggestive of an independent borderline effect in decreasing the SD duration (p = 0.04). The latter is consistent with previous reports of the shortening of the QT interval for LQTS3 following β-adrenergic stimulation. This contrasts with the known effects of β-adrenergic stimulation in increasing QT interval in LQTS1 and LQTS2^39^. Furthermore, dobutamine challenge, age, and genotype exerted no interactive effects on the SD duration.

The QC and QD durations provide indications of total ventricular excitation period including both activation and recovery. Both QC and QD durations were analysed due to the uncertainty in the murine model as to which exact ECG interval corresponds accurately to the human QT interval. The statistical analysis of both the QC and QD durations yielded similar associations, but for which there were higher significance values for QD duration. This would be explicable in terms of the QD duration encompassing more of the timecourse of *I*_Na-L_ than the QC duration. Thus, dobutamine challenge exerted no independent effects on QC and QD duration. However, age independently increased both QC and QD durations (p = 0.03 and p = 0.002 respectively). In addition, genotype independently increased both QC and QD durations (p = 0.001 and p < 0.001 respectively). There were no interactive effects on either QC or QD duration.

Thus, *Scn5a*+*/ΔKPQ* mice consistently showed longer QC and QD durations under all circumstances, consistent with the clinical phenotype. However, in the case of QC duration, this difference was not attributable to changes in SC duration because all differences observed concerning SC duration were not significant. Therefore, the differences observed for QC durations were mainly attributable to differences in activation time, as reflected in QS duration, and not differences in recovery time, as reflected in SC duration. Thus, the results bearing on QS duration indicate interacting effects between age and genotype. This reflects the independent non-interacting effects of age and genotype upon the QC duration. Different inferences could be made from measurements of QD duration. Thus, the significant difference in QD duration appears to arise from (a) the significant effect of genotype on SD duration and (b) the interacting effects of age and genotype on QS duration.

### Histological analysis of fibrosis

**Figure 4:**
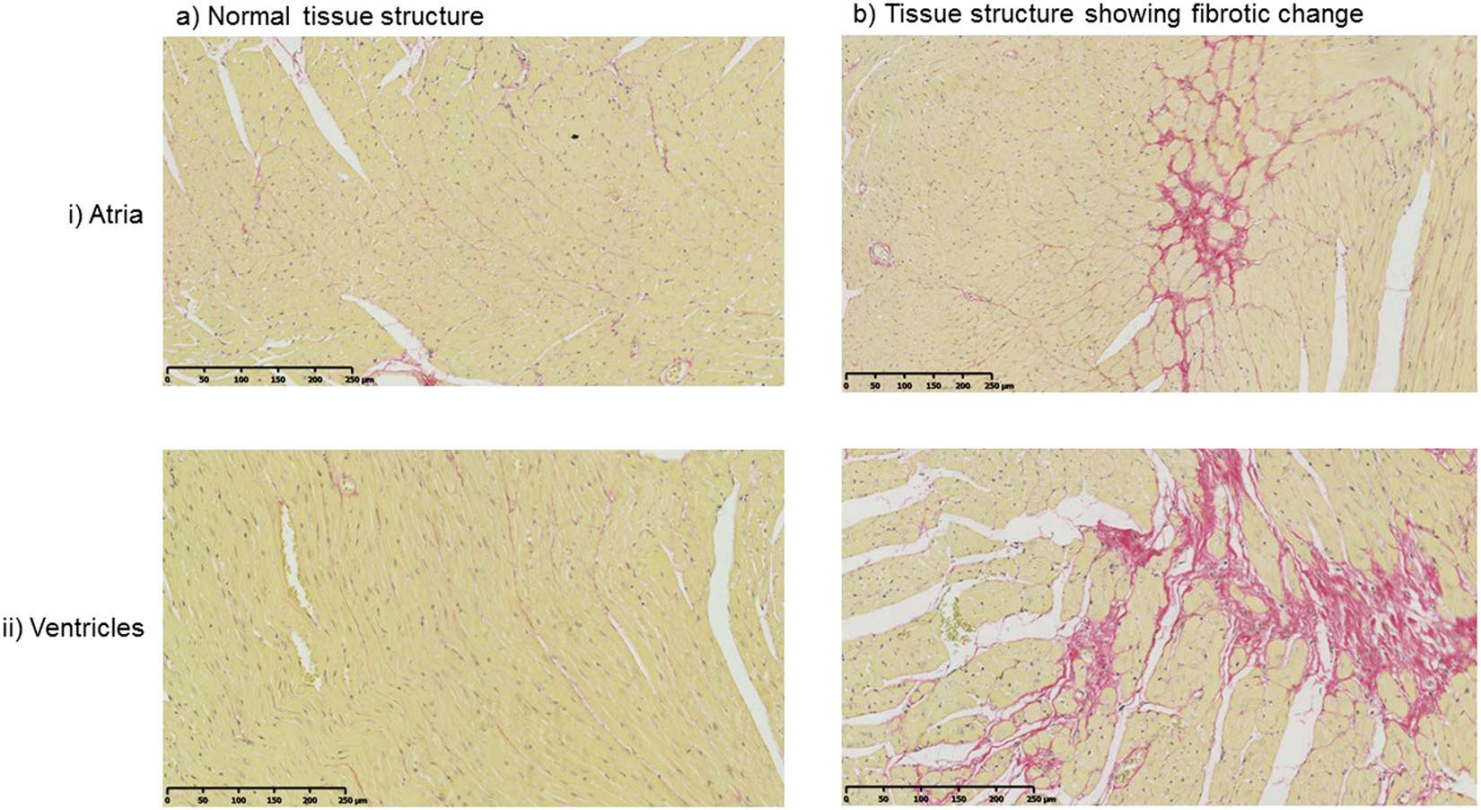
Representative example of the absence of fibrotic change in young WT (**a**) and the presence of fibrotic change in an old Scn5a+/∆KPQ mouse (**b**) showing atrial (i) and ventricular (ii) sections.

We next sought correlations between the above electrocardiographic changes and the absence (Fig. 4a) or presence (Fig. 4b) of fibrotic change in the atria (i) and ventricles (ii) with similarly stratified groups of picrosirius red stained hearts (Fig. 4). Whereas gain of Na^+^ channel function would be expected to increase conduction velocity, fibrotic change may compromise conduction velocity^40^. This may arise through compromised gap junction function^41^ or fibroblast fusion to cardiac myocytes increasing their membrane capacitance^42^. However, the resulting effects on conduction velocity need not show simple correlations with these variables.

**Figure 5:**
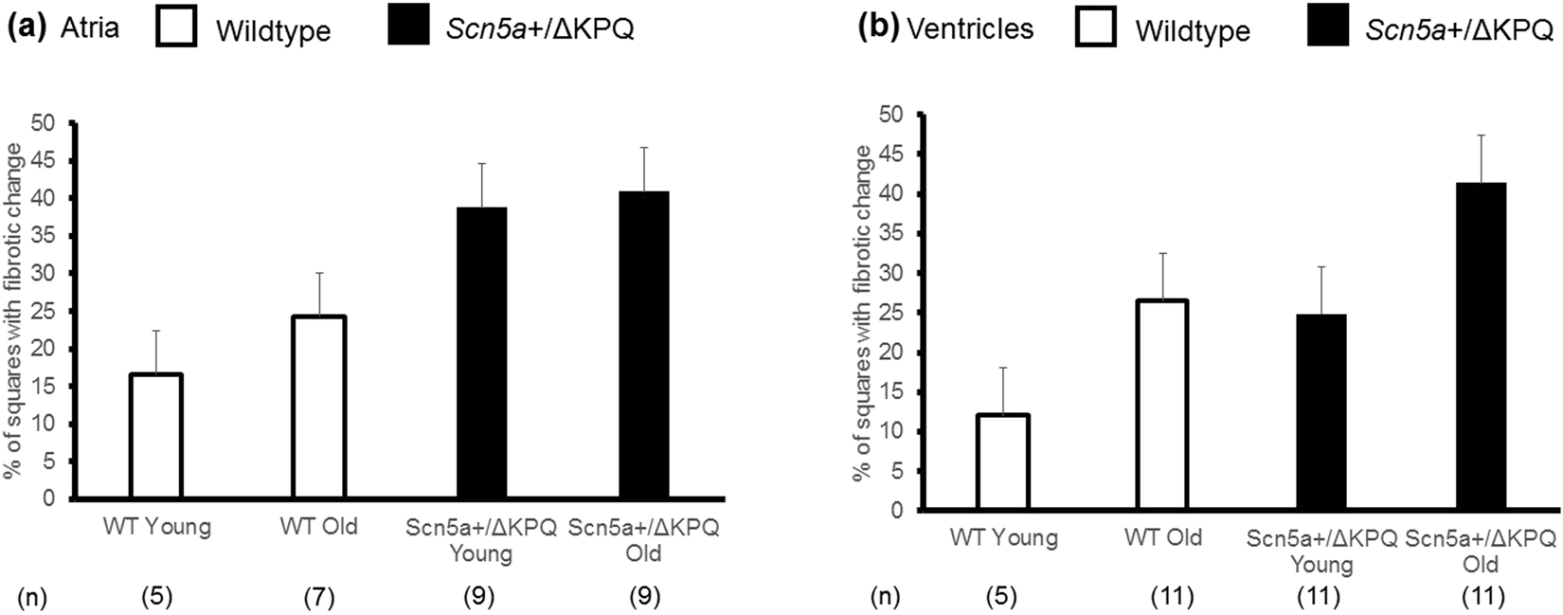
Mean results for the fibrosis in the atria (**a**) and ventricles (**b**) Morphometric squares expressed as a percentage of all squares covering the tissues showing positive evidence of fibrotic changes as detected by picrosirius red staining in the different experimental groups. Numbers of hearts (n) shown below each bar. Linear regression with young and WT as reference categories revealed significant independent effects of (i) age in influencing ventricular (regression coefficient = 16, p < 0.001) but not atrial fibrosis and (ii) *Scn5a*+*/∆KPQ* vs WT genotypes on both atrial (regression coefficient = 19, p < 0.001) and ventricular (regression coefficient = 14, p < 0.001) fibrosis. There were no interacting effects arising from age and genotype.

Histological assessments of the extent of fibrosis were carried out blindly by two investigators independently. Results were then compared for consistency by applying an intra-class correlation coefficient analysis (ICC). The ICC was 0.979 and 0.989 respectively for the atrial and ventricular fibrosis data, indicating a high level of consistency and permitting mean values for both atria (Fig. 5a) and ventricles (Fig. 5b) to be analysed. It was then possible to assess the extent to which the observed fibrotic change was sufficient to alter electrophysiological parameters.

Linear regression was performed to investigate the association between the level of fibrosis with age and genotype in the atria and ventricles. This used young and WT as reference categories. The analysis revealed significant independent effects of (a) age in influencing ventricular (regression coefficient = 16, p < 0.001) but not atrial fibrosis and (b) *Scn5a*+*/∆KPQ* vs WT genotypes on both atrial (regression coefficient = 19, p < 0.001) and ventricular (regression coefficient = 14, p < 0.001) fibrosis. There were no interacting effects arising from age and genotype. Thus, in atrial sections, the *Scn5a*+*/ΔKPQ* genotype but not age independently increased the level of fibrosis (p < 0.001) and there were no interactive effects between these factors. However, both this level of fibrosis and previous reports of reduced Na_v_1.5 expression with age in *Scn5a*+*/*ΔKPQ, appeared insufficient to modify P wave durations, in agreement with an earlier report^9^. Thus, it would appear that a lone association of *Scn5a*+*/ΔKPQ* genotype with increased fibrosis does not suffice to alter P wave duration. For the ventricles, genotype and age independently increased the level of fibrosis (p < 0.001 for both) and there were no interactive effects. Thus, it could be deduced that the independent effects of *Scn5a*+*/ΔKPQ* genotype and old age on increasing the level of fibrotic change match the electrophysiological finding that age and genotype interact to produce reduced conduction in the ventricles, reflected in the prolonged QS duration.

### Incidences of repolarisation alternans

Alternans in the ECG is defined as a presence of alternating changes in waveform amplitude^43^. Alternans reflects electrophysiological instabilities and clinically, repolarisation alternans, specifically T wave alternans, appears to predict clinical arrhythmic events with greater accuracy than other non-invasive markers, such as heart rate variability, signal averaged ECG, or reduced ejection fraction. It has also been implicated in the pathophysiological mechanism of sudden cardiac death (SCD)^43^. Ventricular repolarisation alternans is known to occur in LQTS^44^. However, previous reports have found conditions of adrenergic activity exerted paradoxical effects on the incidence of repolarisation alternans in LQTS3^45–47^.

**Figure 6:**
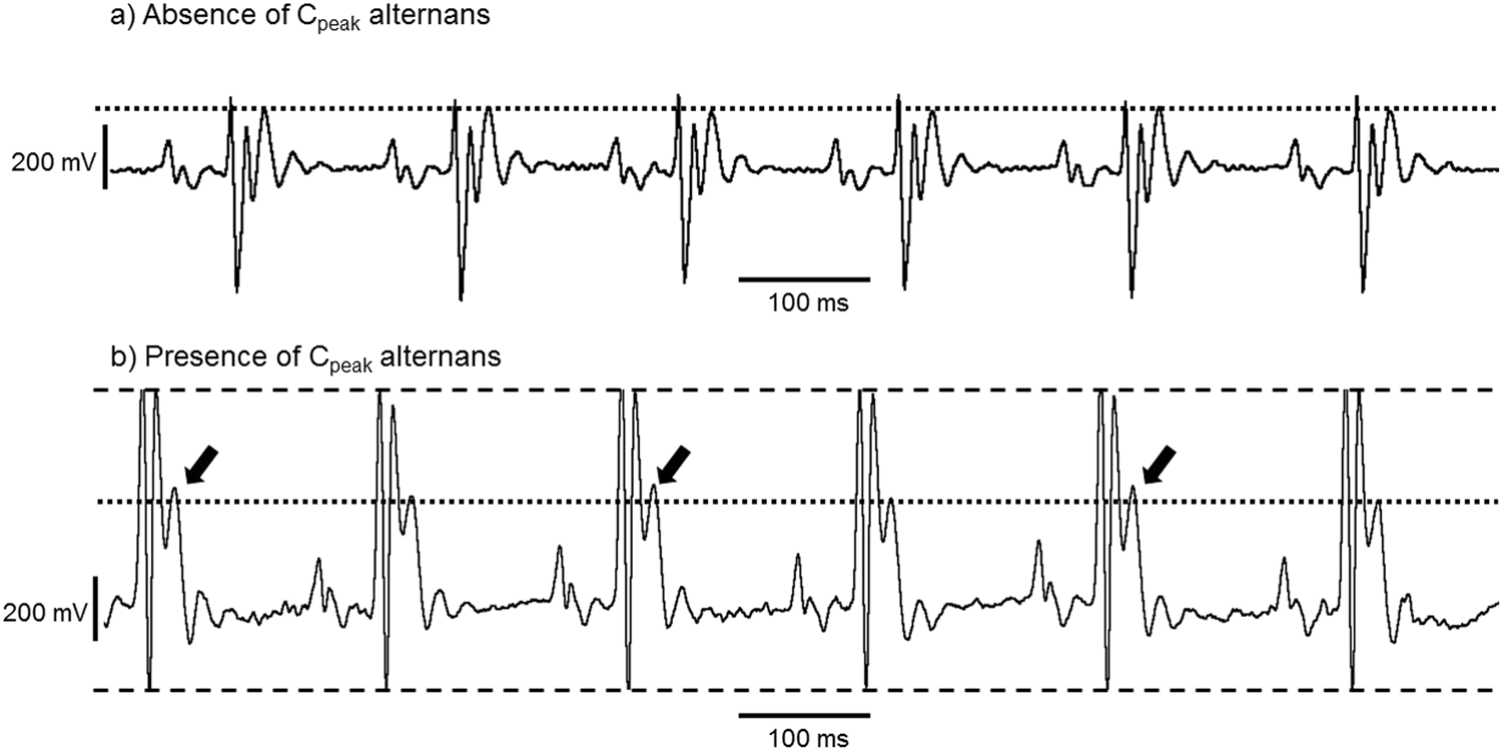
Representative ECG traces to show the absence (**a**) and presence (**b**) of C_peak_ alternans (**a**) This typical trace from a young WT animal shows normal sinus rhythm with the absence of C_peak_ alternans, with the horizontal dotted line superimposed indicating that the level of the C_peak_ remains consistent. (**b**) This typical trace from an old *Scn5a*+/∆KPQ animal shows normal sinus rhythm with the presence of C_peak_ alternans, with the arrows indicating where C_peak_ alternately increases its amplitude higher than the level of the other C_peak_ deflections indicated by the horizontal dotted line. The dashed horizontal lines are to show that the QRS complex amplitude is remains consistent through the recording duration.

The present experiments compared incidences and durations of alternans episodes before and following dobutamine challenge, and any association of the resulting changes with age or genotype. This was accomplished by examining for changes in the amplitude of the largest ECG deflection reflecting ventricular repolarisation, C_peak_ over fixed, 5 min, observation periods both before and following dobutamine challenge (Table 3). Thus, the absence (Fig. 6a) or presence (Fig. 6b) of alternans was assessed from the amplitudes of C_peak_ wave in each consecutive ECG complex normalised to the corresponding amplitude of the C_peak_ in the preceding ECG complex. If the values alternated between being >1 then <1 for a minimum of 12 consecutive beats, then this was counted as alternans. The duration of alternans was assessed from the number of beats over which this occurred. A multilevel negative binomial regression was used to assess association between numbers of incidences and of beats with C_peak_ alternans with respect to dobutamine challenge, age and genotype. This used before dobutamine challenge, young and WT as reference categories respectively. The results showed an independent effect of dobutamine challenge decreasing the incidences of and number of beats with alternans (regression coefficients −2.7 and −3.9 giving significances of p < 0.001 and 0.004 respectively), but no *independent* effects of age or genotype. However, age and *Scn5a*+*/∆*KPQ genotype had a significant *interactive* effect on increasing the incidences of and number of beats with alternans (regression coefficients 3.3 and 6.2 giving p = 0.02 and 0.02 respectively). There were no other significant interactive effects.

**Table 3:** Mean number of incidences and beats with C_peak_ alternans pre-and post-dobutamine challenge.

	(n)	Pre-dobutamine Challenge		Post-dobutamine Challenge	
		*Incidences*	*Beats*	*Incidences*	*Beats*
WT young	(6)	3.7 ± 2.16	50.3 ± 28.35	0.5 ± 0.34	7.2 ± 4.62
WT old	(5)	1.6 ± 1.17	22.0 ± 15.78	0.0	0.0
*Scn5a*+*/KPQ* young	(7)	2.7 ± 2.07	35.3 ± 26.72	0.0	0.0
*Scn5a*+*/KPQ* old	(6)	10.7 ± 5.60	160.7 ± 81.36	1.3 ± 0.99	22.3 ± 17.97

Thus, the present findings showed that age and genotype had an interactive association with a higher incidence and number of beats with C_peak_ alternans indicating the old *Scn5a*+*/ΔKPQ* mice show greater electrophysiological instability. In addition, dobutamine challenge reduced the incidence of repolarisation alternans suggestive of anti-arrhythmic effects. This finding parallels clinical observations that LQTS3 does not share the clearcut pro-arrhythmic features following adrenergic stimulation in other LQTS.

### Delays between increased ventricular rate and ventricular repolarisation change following dobutamine challenge

Previous reports have implicated cellular abnormalities with LQTS, which delay the QT interval shortening that normally follows an increased heart rate. This would result in an increased transient susceptibility to R-on-T related arrhythmias, where activation events are superimposed on a relatively delayed T wave of the preceding beat and this could degenerate into polymorphic VT^48, 49^. These delays are also likely to elude straightforward ECG detection. As with LQTS, the focus of ECG interpretation is generally on the prolongation of QT duration rather than the response of this QT duration to rate changes.

**Figure 7:**
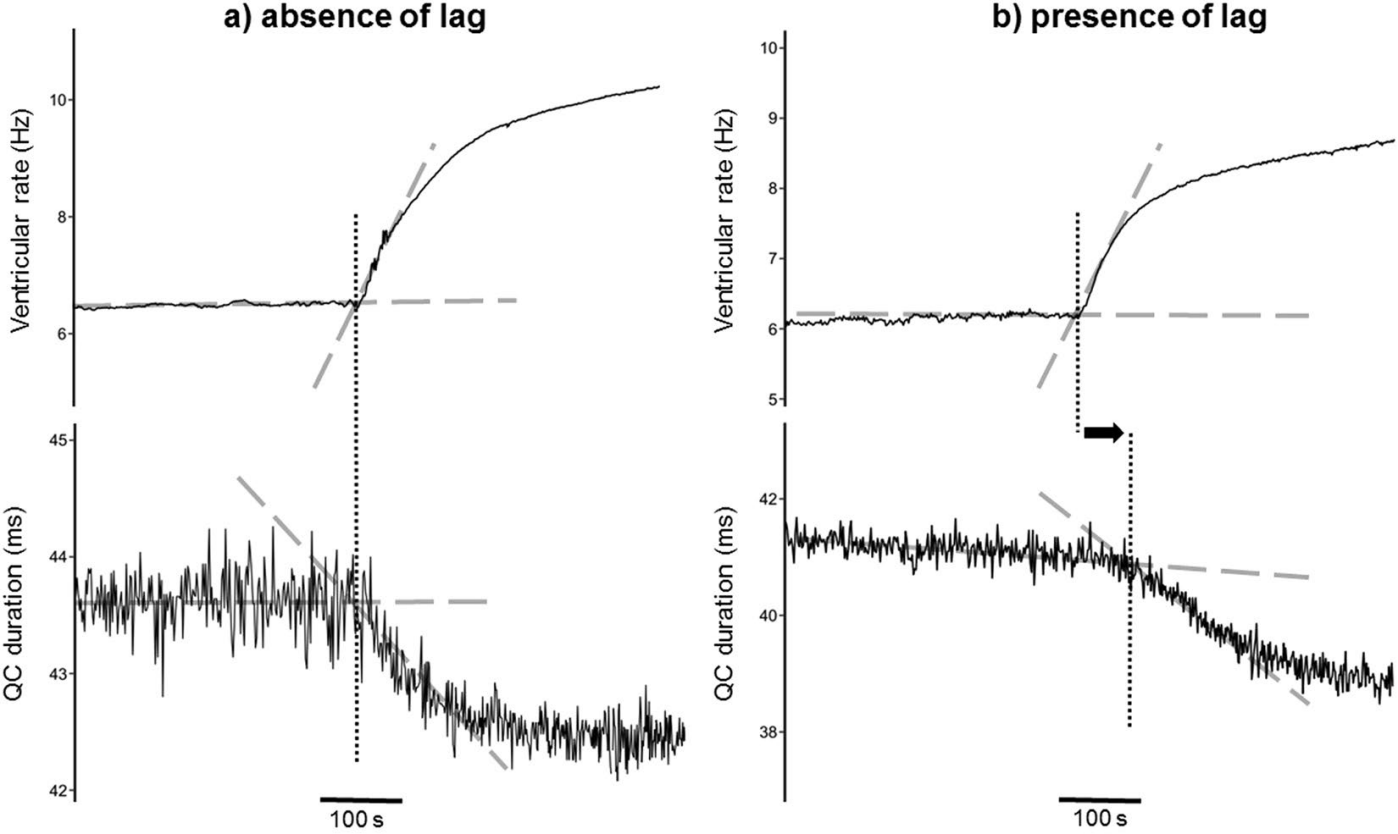
Representative examples showing the absence and presence of the lag in QC duration The dashed lines follow the gradient of the corresponding parts of the traces, with any intersection between two dashed lines indicating a change of gradient and hence an increase or decrease. These changes are marked by the vertical dotted lines. (**a**) These records represent the *absence* of a time lag between the ventricular rate increase (top trace) and the QC duration decrease (bottom trace) following dobutamine challenge exemplified in a young WT mouse. Thus, the vertical dotted line marks the same time point on each trace. (**b**) These records represent the *presence* of a time lag between the ventricular rate increase (top trace) and the QC duration decrease (bottom trace) following dobutamine challenge exemplified in an old *Scn5a* +/∆KPQ mouse. Thus, the vertical dotted lines mark different time points on each trace, with the horizontal arrow indicating this difference.

The present experiments accordingly explored for an absence (Fig. 7a) or presence (Fig. 7b) of such delayed changes in ventricular repolarisation duration following ventricular rate increases produced by dobutamine challenge (Table 4). The time lags between changes in QC and QD intervals and changes in ventricular rate were measured. A logistic regression model was then applied to the number of mice showing such a QC or a QD interval lag. Statistical testing was not applied to length of QC or QD interval lags due to the large number of mice showing no lag and the consequent large standard errors. The present analysis demonstrated an association between *Scn5a*+*/ΔKPQ* and the presence of both a QC and a QD interval lag (regression coefficients = 30 and 36; p = 0.02 and p = 0.004 respectively). Age was a confounding factor only for QC lag (regression coefficient = 8.9; p = 0.08), but this reflected one old WT outlier. Furthermore, as indicated above, it is QD interval that best reflects contributions from *I*_Na-L_. These findings would therefore suggest that delays in changes in repolarisation duration in response to rate changes primarily result from effects of *I*_Na-L_ in turn largely dependent on genotype.

**Table 4:** Number of mice with a time lag between their ventricular rate increase and ventricular repolarisation shortening following dobutamine challenge and the mean length.

	(n)	QC interval lag		QD interval lag	
		*Number*	*Length (ms)*	*Number*	*Length (ms)*
WT young	(7)	0	0.0	0	0.0
WT old	(5)	1	8.0	1	13.0
*Scn5a*+*/KPQ* young	(7)	3	26.4 ± 20.84	6	47.1 ± 21.38
*Scn5a*+*/KPQ* old	(6)	5	50.8 ± 21.54	4	101.7 ± 39.36

## Discussion

The present work extended previous explorations for electrophysiological phenotypes of LQTS3 using electrocardiographic (ECG) methods in intact genetically modified mice carrying the Na_v_1.5 gain-of-function *Scn5a*+*/ΔKPQ* mutation. We extend findings from previous studies through (a) a detailed breakdown of ECG components in terms of their underlying electrophysiological events to demonstrate novel potentially pro-arrhythmic changes in LQTS3. In particular, this revealed age-related changes related to conduction velocity, in addition to the alterations in recovery from excitation previously established in LQTS. (b) The present study demonstrated an underlying age and genotype related fibrotic change for the first time for LQTS3. (c) Furthermore, this study suggested a basis for the paradoxical responses to β-adrenergic stimulation in which we correspondingly demonstrate (d) an increase in the incidence of repolarisation alternans in old S*cn5a*+*/ΔKPQ* mice, which reduces with dobutamine challenge. This demonstrates anti-arrhythmic effects of β-adrenergic stimulation. This was accompanied by (e) slowed responses in ventricular repolarisation adaptation in S*cn5a*+*/ΔKPQ* mice in response to dobutamine challenge, which could highlight a potentially pro-arrhythmic effect during transient heart rate increases.

Firstly, episodes of ventricular electrophysiological activity in each ECG complex were separated into multiple activation and recovery components for the first time in the murine *Scn5a*+*/ΔKPQ* system. This extends previous analysis using only the single indicator of QT interval^9, 28^. Thus, *activation* changes were also demonstrated, extending previous results that only examined *recovery* abnormalities in LQTS models. Two, QC and QD, parameters describing durations of ventricular electrophysiological activity were identified and analysed. Both parameters were prolonged independently both with the presence of *Scn5a*+*/ΔKPQ* genotype and age. Prolongations of the QC duration were attributed to increases in the activation, QS, component, in turn arising from interacting effects of age and *Scn5a*+*/ΔKPQ* genotype. However, neither age nor *Scn5a*+*/ΔKPQ* genotype affected the recovery, SC, component. The SC component is therefore unlikely to include any APD prolongation attributable to the increased *I*_Na-L_ known to occur in *Scn5a*+*/ΔKPQ* ^18^.

In contrast, prolongation of the QD duration arose from changes in *both* activation, QS, *and* recovery, SD, components. Thus, SD was prolonged by the *Scn5a*+*/ΔKPQ* genotype but not by age. SD is therefore likely to include APD components specifically related to the increased *I*_Na-L_ in *Scn5a*+*/ΔKPQ*. A fuller breakdown of clinical ECG QT duration into activation and recovery components, than that currently adopted, could therefore reveal that prolonged QT intervals in LQTS reflect not only recovery, but also activation abnormalities.

This combination of both activation and recovery abnormalities observed in the old *Scn5a*+*/ΔKPQ* mouse might then account for an appearance of *overlap syndromes* with age in LQTS3 patients. These syndromes are characterised by a co-existence of more than one of the separate phenotypes expected from individual *SCN5A* mutations in the same patient^50^. *SCN5A* mutations are variously associated with LQTS3, BrS, progressive cardiac conduction defect (PCCD), sick sinus node syndrome (SSS), atrial fibrillation (AF), and even dilated cardiomyopathy (DCM)^51^. We thus complement previous reports in which an age-dependent penetrance of ECG characteristics was associated with the *SCN5A*-*1795insD* mutation. The carriers of this particular mutation showed QT-prolongations and signs of conduction disease both of which were present from birth. They also showed BrS features that developed at a later age^52, 53^. The presence of overlap syndromes in ageing LQTS3 patients could have potential implications concerning the choice of therapeutic agents.

Secondly, the present experiments went on to explore for possible structural mechanisms for the observed age-dependent phenotypic changes. They complement the previous studies in murine *loss*-of-function *Scn5a*+*/*− hearts that had implicated fibrotic change in the arrhythmogenesis in BrS,^40^. The present study associated for the first time the *gain*-of-function *Scn5a*+*/ΔKPQ* mutation with increased fibrosis in both atria and ventricles. The observed increases in fibrotic change likely arise from different signalling cascades from those in the loss-of-function BrS murine model, where Na^+^ channel deficiency manifests as a TGF-β1-mediated fibrosis associated with *Scn5a* disruption or aging^54^. For example, the mechanism in the gain-of-function *Scn5a*+*/ΔKPQ* could relate to altered Ca^2+^ homeostasis accompanying the elevated *I*_Na-L_^55^. The resulting Ca^2+^ load could then compromise mitochondrial function, increasing a pro-fibrotic ROS production^56, 57^.

Furthermore, the observed independent actions of both age and genotype upon ventricular fibrotic change correlated with the electrophysiological interacting effects of age and *Scn5a*+*/*ΔKPQ genotype in compromising ventricular activation. The mechanistic basis for this could potentially involve gap junction disruption from the fibrotic change, which would then increase resistance to local circuit currents and hinder AP propagation^41^. Alternatively, fibroblasts or myofibroblasts (Mfbs) could form couplings with cardiomyocytes through Cx43 and Cx45 binding^58, 59^. The resulting increased cell capacitance would reduce conduction velocity to extents varying with ratio of the numbers of Mfbs to cardiomyocytes^42, 60, 61^. This would increase arrhythmic tendency through formation of slow-conducting re-entry circuits^62, 63^. Thus, therapeutic agents for LQTS3 patients, particularly with age, may need to addresses the ventricular activation deficit. One possibility may be through targeting the fibrotic change itself. For example, the use of angiotensin-converting enzyme inhibitors to inhibit RAAS have been shown to reduce myocardial fibrosis in both experimental and clinical studies^64, 65^.

Thirdly, ECG measurements were compared before and following acute β-adrenergic challenge. Thus dobutamine challenge exerted positive chronotropic and dromotropic effects, reflected in increased ventricular rates and reduced PR segment and PR interval durations, whilst leaving QS intervals unchanged. This could reflect similar and positive effects of β-adrenergic stimulation upon both pacing currents and atrial but not ventricular conduction in both WT and *Scn5a*+*/ΔKPQ*.

Fourthly, ventricular repolarisation alternans was compared between experimental groups, and the effect of dobutamine challenge upon this then investigated in the study population. Before pharmacological intervention, old *Scn5a*+*/ΔKPQ* mice showed higher incidences and durations of ventricular repolarisation alternans. Clinically, ventricular repolarisation alternans, reflected in T-wave alternans (TWA), is known to occur in LQTS^44^, with implications for the pathophysiological mechanism of sudden cardiac death (SCD)^43^. Repolarisation alternans is closely related to the Ca^2+^ cycling and mechanical alternans^43^ attributed to variations in intracellular sarcoplasmic reticulum (SR) Ca^2+^ release^66^. Thus, both are abolished by reduced [Ca^2+^]_o_ or ryanodine-mediated RyR2 block^44^. An increased *I*_Na-L_ attributed to *Scn5a*+*/ΔKPQ* would increase [Ca^2+^]_i_ through the power function relationship it bears to [Na^+^]_i_^67, 68^ thereby contributing to development of alternans. The observed interacting effects of *Scn5a*+*/ΔKPQ* genotype and age are consistent with increased incidences of alternans in old *Scn5a*+*/ΔKPQ* mice producing an underlying Ca^2+^ cycling alternans given age-related reductions in myocardial SR Ca^2+^ ATPase (SERCA) density^69^.

Dobutamine challenge then exerted anti-arrhythmic effects in reducing the incidence and duration of repolarisation alternans. Clinical findings however indicate that adrenergic activity and heart rate exert paradoxical effects on the incidence of TWA in LQTS3. This is reflected in earlier findings of nocturnal episodes of peak TWA in LQTS3 patients that were associated with conditions of rate suppression^45, 46^. Studies separating the effects of β-adrenergic stimulation and imposed heart rates implicated the increases in heart rate rather than β-adrenergic stimulation in TWA^47^. The present finding that β-adrenergic stimulation paradoxically reduces repolarisation alternans in LQTS3 might reflect reduction of the normal phospholamban (PLB)-mediated inhibition of SERCA-mediated Ca^2+^ re-uptake^70, 71^. Alternatively, β-adrenergic stimulation has been reported to shift the *I*_Na_ inactivation–voltage relationship to more negative potentials decreasing window current and hence I_Na-L_^72–75^. This would decrease [Na^+^]_i_, and in turn [Ca^2+^]_i_ thereby possibly reducing repolarisation alternans.

Finally, the experiments demonstrated that with the *Scn5a*+*/ΔKPQ* genotype, there was a delay in the onset time over which ventricular repolarisation duration changed following corresponding increases in ventricular rate produced by dobutamine challenge. In response to a change in ventricular rate, the QT interval normally shortens immediately and then follows a more gradual mono-exponential reduction to a new steady-state value^76^. Thus, a discrepancy in the onset time of this shortening could result in a transient increase in ventricular repolarisation time relative to RR interval. This may confer arrhythmic susceptibility during heart rate increases in LQTS3 patients for example^48^. The present findings thus complement previous reports that had similarly reported paradoxically increased AP duration in mice modelling LQTS3 following sudden rate increases^18^. The previous studies were performed in isolated ventricular preparations under artificial pacing rather than employing intact animals with normal sympathetic innervations and β-adrenergic stimuli replicating the *in vivo* situation.

## Conclusions

The present study demonstrates for the first time that a gain-of-function *Scn5a*+*/ΔKPQ* mutation is associated with an increased level of fibrotic change. This increased fibrosis associated with *Scn5a*+*/ΔKPQ* and age slows conduction in the ventricles and could be the cause of the prolonged activation, QS, interval in old *Scn5a*+*/ΔKPQ* mice. This has clinical implications in that arrhythmic events in elderly LQTS patients may be due to an activation abnormality as opposed to a sole recovery abnormality. Thus, therapeutic options for LQTS patients may need to be expanded from solely treating the prolonged ventricular repolarisation to treating any activation deficits arising from fibrotic change. Furthermore, therapeutic β-adrenergic agonists or antagonists for LQTS3 patients specifically should consider the paradoxical effects of such administration. The present study demonstrates both pro- and anti-arrhythmic effects of dobutamine challenge, recapitulating the clinical observations of its paradoxical effects. However, the present results suggest that LQTS3 patients are likely to be pro-arrhythmic during the change of heart rate following β-adrenergic stimulation. Moreover, the present results suggest that the anti-arrhythmic effects of β-adrenergic stimulation may be more apparent with age, as there is a reduction in repolarisation alternans and improvement in conduction following acute dobutamine administration. This indicates that age-specific drug choice may be required as the disease phenotype can change with age.
